# Genome-Wide Association Study of First-Parity Reproductive Traits in Suzi Pig

**DOI:** 10.3390/genes16111335

**Published:** 2025-11-06

**Authors:** Yanfeng Fu, Weining Li, Chaohui Dai, Chao Liao, Jinhua Cheng, Hui Li, Weimin Zhao

**Affiliations:** Institute of Animal Science, Jiangsu Academy of Agricultural Sciences/Key Laboratory for Crop and Animal Integrated Farming, Ministry of Agriculture and Rural Affairs/Jiangsu Province Engineering Research Center of Precision Animal Breeding, Nanjing 210014, China; liwn@jaas.ac.cn (W.L.); chdai1993@163.com (C.D.); lcsolar@163.com (C.L.); 20090016@jaas.ac.cn (J.C.); lhlydk@126.com (H.L.); zhao_weimin1983@aliyun.com (W.Z.)

**Keywords:** black pig, GWAS, first-parity, litter size, heritability, GO

## Abstract

Backgrounds: Objective of this study is to conduct a genome-wide association study (GWAS) of first-parity reproductive traits in Suzi pigs to identify significant single-nucleotide polymorphisms (SNPs) or candidate genes influencing these traits. Methods: This research employed technologies including the Zhongxin 50K SNP chip, simplified genome sequencing, resequencing, and the 100K SNP liquid chip to perform genome-wide SNP detection on 898 Suzi sows. Genotype data and phenotypic data were combined to do GWAS, gene annotation, and enrichment analysis. Results: Results showed that this study obtained phenotypes of 33 first-parity reproductive traits from 574 sows. GWAS results indicated there were 10 first-parity reproductive traits significantly associated with SNPs, and these traits were AFS, AFF, NNB, NH, NW, NS, NM, ND, PB, and CCN. These 10 traits were significantly associated with 60 SNPs, with 15 (25%) located on chromosome 2-the highest proportion. The SNPs significantly associated with AFS and AFF were largely identical. Genome-wide variance component analysis revealed that among the 10 traits with significantly associated SNPs in GWAS, there were 5 traits that exhibited genome-wide heritability ≥ 0.01. Trait of NM showed the highest heritability (0.65–0.7). These significantly associated SNPs annotated 20 candidate genes, including ADAMTS19, PROP1, ZNF354B, PCARE, LUZP2, VIRMA, EPHA5, AAAS, SLCO3A1-SV2B, KIF18A-BDNF, SERGEF, DYNLRB2, HNF4G, CATSPERD, HSD11B1L, DICER1, RARG, PCDHAC2, KRT79, and HSD17B2. GO analysis of candidate genes revealed that the top three biological processes were cell adhesion, positive regulation of cell projection organization, and positive regulation of neuron projection development. KEGG results showed the top three pathways were inositol phosphate metabolism, glutamatergic synapse, and phosphatidylinositol signaling system. Conclusions: These findings provide a foundation for the reproductive breeding of Suzi pigs and offer new insights into biological breeding in pigs.

## 1. Introduction

The native pig breeds in China are recognized for their excellent reproductive traits, high-quality meat, and strong stress tolerance [1]. However, the native black pig breeds in China exhibit inferior litter size, growth rates, feed efficiency and lean meat percentage compared to commercial pig breeds, such as Yorkshire [2,3]. So, Chinese pig breeders usually developed a new pig breed by crossbreeding Chinese native pigs and commercial pigs, which could combine the advantages of both pigs, such as Jinwu pigs by crossing Jinhua pigs with Duroc pigs [4]. In this study, the experimental animals are one of Chinese black pigs, called Suzi pig, which is a new breed been cultivated for more than 14 years and is a breed to balance reproductive traits and growth rates. Suzi pig were developed by crossing Suzhong pig (approved as a Jiangsu province’s new breed in 2001) [5], Huai pig (one of Chinese native pig breeds) [6] and Berkshire pig (one of commercial pig breeds) [7]. Suzi pigs has more than 60% Chinese native bloodline, making it a high-quality black pig breed.

Reproductive traits are critical economic and breeding characteristics, with their performance levels directly impacting the productivity of breeding sows [8]. The age at first service (AFS), total number of piglets at birth (TNB), number of piglets born alive at birth (NBA), number of stillborn piglets (NS), number of piglet deformities (ND), and piglets mummified at birth (NM) are key indicators for evaluating sow reproductive performance [9]. The heritability of TNB or NBA in sows is approximately 0.1, classifying it as a trait with low heritability [10]. Former research findings indicated that traditional breeding methods yielded relatively slow progress in improving sow litter size performance [11]. With advances in biotechnology, genome-wide association studies (GWAS) have begun playing a significant role in animal and plant breeding [12].

During the last ten years, genome-wide association studies (GWAS) have repeatedly identified quantitative trait loci (QTL) for litter traits on Sus scrofa chromosomes (SSC) 1, 7, 8, 11, and 14 in Yorkshire sows [13]. Fine-mapping with whole-genome sequence (30–40×) has narrowed several critical intervals to <0.5 Mb, enabling the prioritization of candidate genes such as ESR1, PGR, RBP4, and ARF6 that regulate ovulation rate, uterine receptivity, and conceptus survival [14]. The objective of this study is to identify significant associated SNPs and their annotated candidate genes for first-parity reproductive traits in Suzi pigs through GWAS analysis. This research seeks to further enhance the reproductive performance and breeding efficiency of this Chinese black pig breed, while providing technical support for promoting efficient breeding of high-quality pigs.

## 2. Materials and Methods

### 2.1. Experimental Animals

The experimental animals were Suzi pig—a breed under cultivation. These animals were fed in Experimental Pig Farm at the Liuhe Base of the Jiangsu Academy of Agricultural Sciences (Nanjing, Jiangsu, China. Before July 2021) and the Pig Farm of Jiangsu Ronggang Breeding Pig Co., Ltd. (Taixing, Jiangsu, China. After July 2021). The experimental period (age at first farrowing of sows) spanned from December 2017 to April 2025. Whole-genome sequencing was performed on 898 Suzi pigs. Excluding gilts, boars, and castrated boars, 574 sows had complete first-parity reproductive performance records.

### 2.2. Measurement of Reproductive Performance

All experimental sows underwent comprehensive first-parity reproductive performance evaluations. These assessments encompassed 33 reproductive traits, including: age at first service (AFS), age at first farrowing (AFF), comprehensive estimated breeding value (cEBV), gestation period (GP), comprehensive number of piglets born (CNB), total number of piglets born (TNB), number of piglets born alive at birth (NBA), number of non-viable piglets born (NNB), number of healthy offspring (NH), number of weak offspring (NW), number of stillborn piglets (NS), number of mummified piglets (NM), number of deformed piglets (ND), number of male piglets born alive at birth (mNBA), number of female piglets born alive at birth (fNBA), litter birth weight (LBW), individual birth weight (IBW), number of left teats in piglets (NLT), number of right teats in piglets (NRT), total number of teats in piglets (TNT), number of spotted piglets (NP), percentage of pure black piglets (PB), weaning age (WA), number of weaned piglets (NW), individual weaning weight (IWW), litter weaning weight (LWW), daily gain from birth to weaning (DGBW), age at transfer to nursery (AN), body weight at transfer to nursery (BWN), body length at transfer to nursery (BLN), chest circumference at transfer to nursery (CCN), abdominal circumference at transfer to nursery (ACN), and leg circumference at transfer to nursery (LCN).

### 2.3. Sample Collection and Genomic DNA Extraction

Samples were collected from the ear tissue of each experimental pig. After collection, samples were placed in centrifuge tubes containing 70% ethanol for storage. The tubes were stored in dry ice for short-term preservation and transferred to a −20 °C freezer upon return to the laboratory for genomic DNA (gDNA) extraction. gDNA extraction was performed using the Tiangen Animal Blood/Cell/Tissue Genomic DNA Extraction Kit (Tiangen, Beijing, China), following the kit’s protocol. The extracted genomic DNA was stored in centrifuge tubes containing TE buffer.

### 2.4. Whole-Genome Snp Detection

Following quality control verification, genomic DNA was subjected to whole-genome SNP detection in pigs. Detection methods included the Zhongxin 50K SNP chip (462 animals), simplified genome sequencing (206 animals), resequencing (30 animals), and the 100K SNP liquid chip (200 animals). Primary detection instruments included the Illumina Chip Scanner, Illumina HiSeq X Ten genome sequencer, BGI DNBSEQ-T7 gene sequencer, and Boridi series instruments (high-throughput automated nucleic acid extraction platform, fully automated pipette tip array robot, fully automated sample processing system, high-throughput liquid workstation) (Boridi, Shijiazhuang, Hebei, China). Taking Zhongxin 50K chip sequencing as an example, the procedure is as follows. Firstly, adjust the gDNA concentration of all samples to 50 ng/L. Secondly, perform whole-genome amplification on all samples, incubating at 37 °C for 20–24 h. Thirdly, fragment, precipitate, and resuspend the gDNA in hybridization buffer. Fourth, add the resuspended DNA fragments to the chip for hybridization, incubating at 48 °C for 16–24 h. Fifthly, wash to remove non-specifically bound DNA, leaving specifically bound sites for single-base extension. Lastly, scan using the Illumina iScan Reader after staining.

### 2.5. Genome-Wide Association Study (Gwas)

For phenotypic data processing, this study utilized software including ACCESS 2021, EXCEL 2021, and UltraEdit 25.00.0.82 . For genotypic data processing, Plink 4.3.1 software was employed, with custom programs developed to edit and convert genotype data from letter-based to numeric formats. Subsequently, in the R-studio platform, R code was written to call upon packages including ASReml, ASRgwas, openxlsx, data.table, tidyverse, qqman, and RColorBrewer for genotype data cleaning, genome-wide association analysis, variance component estimation, and heritability estimation [15]. Genotype data cleaning involved linking genotype, phenotype, and map data while performing quality control. The quality control standards in this study were as follows: minimum allele frequency (MAF) > 0.05, marker missing rate < 0.2, sample missing rate < 0.2, heterozygosity ≤ 0.8, mean inbreeding coefficient (Fis) < 0.98, and default imputation (missing data is permitted).

The GWAS model employs a mixed linear model (MLM) for continuous traits [16], expressed as y = Xα + Zβ + Wμ + e. Here, y represents the phenotypic value of the trait; Xα denotes the PCA covariates (or Q covariates), constituting fixed effects; Zβ represents SNP effect values, constituting fixed effects; Wμ denotes the kinship matrix, constituting random effects; and e denotes the residual effect. The default *p*-value threshold is 5 × 10^−4^, and variance components are estimated using the P3D method.

Given that association analyses often involve large SNP datasets prone to false positives, multiple testing correction is essential. Three methods are currently used to detect genome-wide significance: Bonferroni correction, FDR, and permutation testing. This study employed the most widely used Bonferroni correction. The threshold in Bonferroni correction was set to “1/(number of SNPs analyzed)”. This threshold typically filters out most false positives. Subsequently, SNPs with *p*-values below this threshold are selected, and finally visualized using a Manhattan plot and QQ plot.

## 3. Results

### 3.1. Results of First-Parity Reproductive Performance

There were 574 experimental animals (Suzi sows) with 33 first-parity reproductive performance records (phenotypes), and analysis of these records yielded the results shown in Table 1. Results indicate that the average AFS was 345.36 days, with a minimum of 141 days; the average AFF was 459.17 days, with a minimum of 256 days; the average cEBV exceeded 100; and the average GP was 113.8 days, with a median of 114 days.

For sows, the average comprehensive litter size (TNB × 50% + NBA × 50%) was 9.34, with a median of 9.5; the average TNB was 9.79, median 10, maximum 16, minimum 3; the average NBA was 8.9, median 9, maximum 16, minimum 1; the average NNB was 0.77, with a maximum of 7, and a minimum of 0. NNB equals the sum of NS, NM, and ND. Among NNB, NS accounted for the highest proportion, followed by NM, while ND was the least proportion (Table 1).

For birth piglets, NBA equals the sum of mNBA and fNBA, and mNBA (4.69) was slightly higher than fNBA (4.41). The average LBW was 10.8 kg, and the average IBW was 1.21 kg. The NLT and NRT were approximately 7.2, indicating an even nipple distribution and a high TNT of 14.51. TNT equals the sum of NLT and NRT. The average NP was around 3, and the median PB was 82%. In 2025, the PB had increased to 93% (Table 1).

For weaned piglets, the average WA was 28.32 d, with a median of 28 d, a maximum of 31 d, and a minimum of 25 d. The average NW was 8.73, with a median of 9, a maximum of 14, and a minimum of 1. The average LWW was 56.34 kg, with an average IWW of 6.55 kg (range: 5.7–9.5 kg). The average DGBW was 207.19 g/d (Table 1).

At transfer to nursery, the average AN was 34.86 d, with an average BWN of 8.61 kg, average BLN of 48.32 cm, average CCN of 43.33 cm, average ACA of 39.62 cm, and average LCN of 10.78 cm (Table 1).

### 3.2. Genome-Wide Association Study

**Figure 1:**
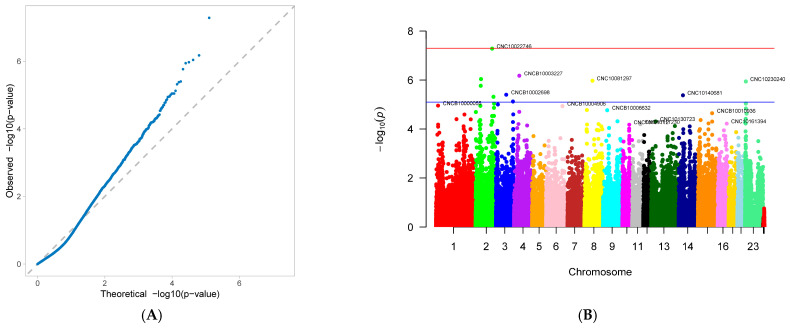
Genome-wide association study results for age at first service (AFS) in Suzi sows: (**A**) QQ plot, (**B**) Manhattan plot.

**Figure 2:**
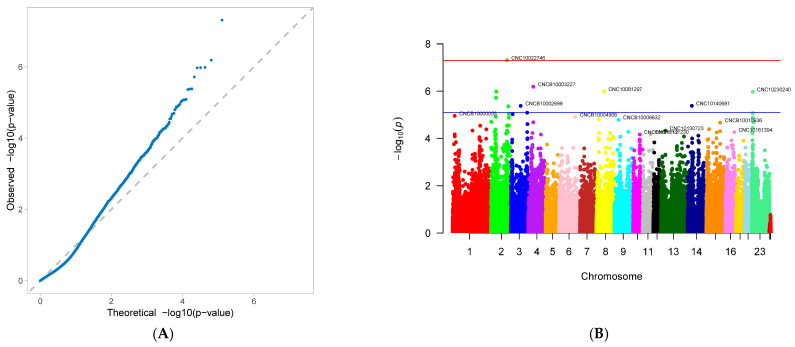
Genome-wide association study results forage at first farrowing (AFF) in Suzi sows: (**A**) QQ plot, (**B**) Manhattan plot.

**Figure 3:**
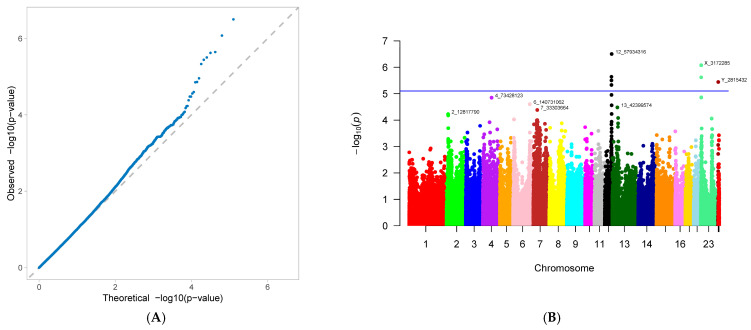
Genome-wide association study results for number of non-viable piglets born (NNB) in Suzi sows: (**A**) QQ plot, (**B**) Manhattan plot.

**Figure 4:**
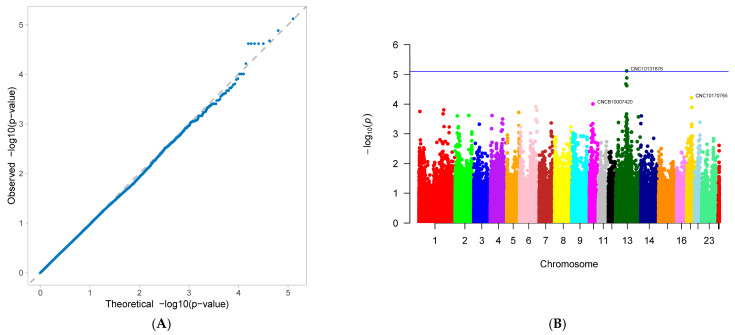
Genome-wide association study results for number of healthy offspring (NH) in Suzi sows: (**A**) QQ plot, (**B**) Manhattan plot.

**Figure 5:**
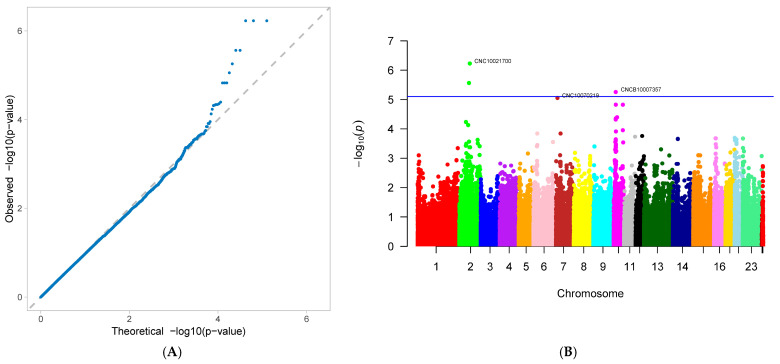
Genome-wide association study results for number of weak offspring (NW) in Suzi sows: (**A**) QQ plot, (**B**) Manhattan plot.

**Figure 6:**
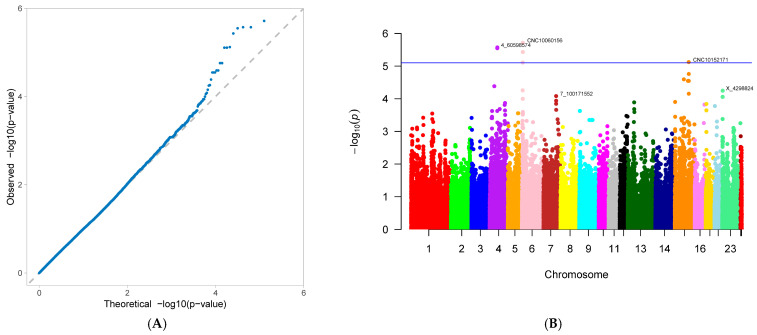
Genome-wide association study results for number of stillborn piglets (NS) in Suzi sows: (**A**) QQ plot, (**B**) Manhattan plot.

**Figure 7:**
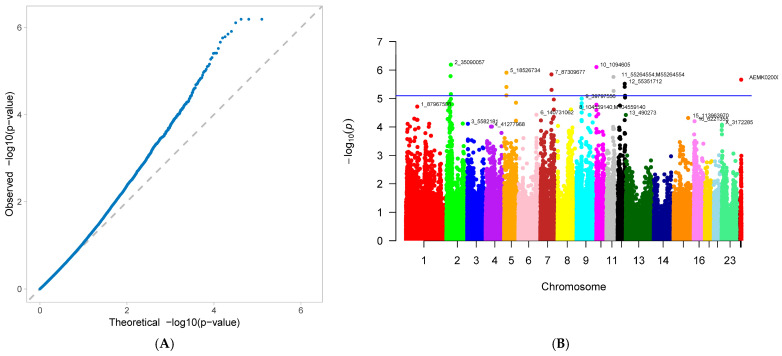
Genome-wide association study results for number of mummified piglets (NM) in Suzi sows: (**A**) QQ plot, (**B**) Manhattan plot.

**Figure 8:**
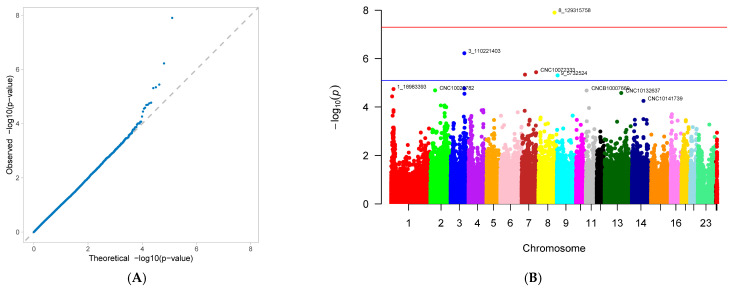
Genome-wide association study results for number of deformed piglets (ND) in Suzi sows: (**A**) QQ plot, (**B**) Manhattan plot.

**Figure 9:**
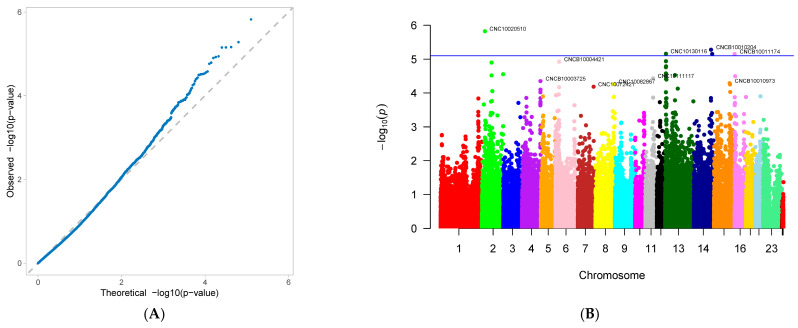
Genome-wide association study results for percentage of pure black piglets (PB) in Suzi sows: (**A**) QQ plot, (**B**) Manhattan plot.

**Figure 10:**
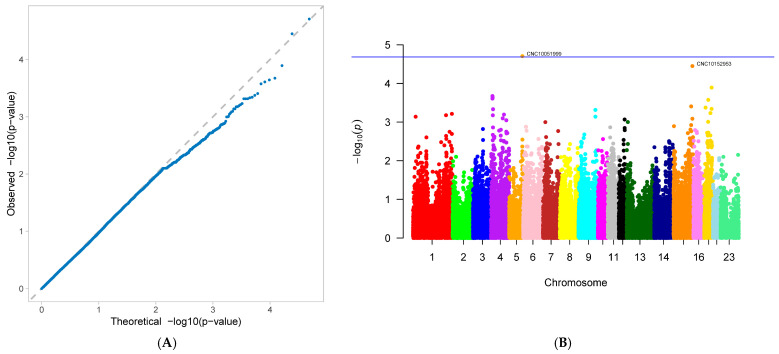
Genome-wide association study results for chest circumference at transfer to nursery (CCN) in Suzi sows: (**A**) QQ plot, (**B**) Manhattan plot.

A genome-wide association study (GWAS) was conducted on 33 first-parity reproductive traits (phenotypes) of Suzi pigs. These traits are associated with 126,606 SNPs, with the effective SNPs ranging from 48,482 to 126,606. GWAS results showed that 10 traits were identified with significantly associated SNPs. These traits were as follows: AFS, AFF, NNB, NH, NW, NS, NM, ND, PB, and CCN. Each trait has one GWAS result, and one GWAS result includes two figures: (A) Quantile-Quantile Plot (QQ-Plot), and (B) Manhattan plot (Figure 1A,B, Figure 2A,B, Figure 3A,B, Figure 4A,B, Figure 5A,B, Figure 6A,B, Figure 7A,B, Figure 8A,B, Figure 9A,B and Figure 10A,B).

Figure 1A, Figure 2A, Figure 3A, Figure 4A, Figure 5A, Figure 6A, Figure 7A, Figure 8A, Figure 9A and Figure 10A are QQ-Plots (Quantile-Quantile Plots), a graphical method for comparing two probability distributions. QQ-plots in Figure 1A, Figure 2A, Figure 3A, and Figure 8A exhibit the characteristic steep upward slope in the latter half, indicating highly satisfactory GWAS analyses for these figures. Figure 4A exhibits a small upward-left spike in the latter half, while Figure 5A, Figure 6A, Figure 7A, and Figure 9A each display a large upward-left spike in the latter half. Figure 10A initially falls below the median line, with two points ultimately positioned on the median line. Figure 1B, Figure 2B, Figure 3B, Figure 4B, Figure 5B, Figure 6B, Figure 7B, Figure 8B, Figure 9B and Figure 10B are Manhattan plots, which plot the *p*-values of all SNP loci across the genome from left to right following GWAS analysis. Figure 1B, Figure 2B, Figure 3B, Figure 4B, Figure 5B, Figure 6B, Figure 7B, Figure 8B, Figure 9B and Figure 10B sequentially identified 10, 9, 7, 1, 6, 8, 17, 5, 5, and 1 significantly associated SNPs. The traits corresponding to Figure 1, Figure 2, Figure 3, Figure 4, Figure 5, Figure 6, Figure 7, Figure 8, Figure 9 and Figure 10 are in sequence: AFS, AFF, NNB, NH, NW, NS, NM, ND, PB, and CCN (Figure 1A,B, Figure 2A,B, Figure 3A,B, Figure 4A,B, Figure 5A,B, Figure 6A,B, Figure 7A,B, Figure 8A,B, Figure 9A,B and Figure 10A,B).

SNPs for each trait were ranked by significance from highest to lowest. For the trait in Figure 1 (AFS), significantly associated SNPs were as follows: CNC10022746, CNCB10003227, CNCB10001752, CNC10081297, CNC10230240, CNCB10001743, CNCB10002698, CNC10140681, CNC10022920, and CNC10032654. For the trait in Figure 2 (AFF), significantly associated SNPs were as follows: CNC10022746, CNCB10003227, CNC10081297, CNCB10001752, CNC10230240, CNCB10001743, CNC10140681, CNCB10002698, and CNC10022920. For the trait in Figure 3 (NNB), significantly associated SNPs were as follows: 12_57934316, X_3172285, 12_56124655, X_3176453, 12_56141636, Y_2815432, and 12_56118044. Figure 4 shows the trait of NH, with only one significantly associated SNP: CNC10131876. Figure 5 shows the trait of NW, with significantly associated SNPs in the following order: CNC10021700, CNC10021702, CNC10021703, CNC10021544, CNC10021546, and CNCB10007357. Figure 6 shows the trait of NS, with significantly associated SNPs in the following order: CNC10060156, 4_60598574, 4_60633363, 4_60143356, CNC10060142, CNC10152171, CNCB10004308, and CNCB10004309. Figure 7 shows the trait of NM, with significantly associated SNPs in the following order: 2_35090057, 2_35184348, 2_35304516, 10_1094605, 5_18526734, 7_87309677, 2_32394198, 11_55264554, M55264554, 1_608863, 12_55351712, 12_55045321, 12_55094012, 5_18448981, 7_88172698, 11_56289609, 2_34903944, and 5_18115698. Figure 8 shows the trait of ND, with significantly associated SNPs being the following: 8_129315758, 3_110221403, CNC10072333, CNC10070593, and 9_5732524. Figure 9 shows the trait of PB, with significantly associated SNPs in the following order: CNC10020510, CNCB10010204, CNC10130116, CNCB10010264, and CNC10011174. Figure 10 represents the trait of CCN, with only one significantly associated SNP: CNC10051999 (Table 2).

These 10 traits are collectively associated with 69 SNPs. Among them, 9 SNPs were common to traits in Figure 1 and Figure 2. After deduplication, these traits shared 60 SNPs. Of these 60 SNPs, 15 were located on chromosome 2, the highest number; seven are located on chromosome 12, the second highest number; four are distributed across chromosomes 4, 5, 6, and 7; three are found on chromosomes 3, 14, and 23; two are present on chromosomes 8, 10, 11, and 13; and one is distributed across chromosomes 9, 15, 16, 24 (X chromosome), and 26 (mitochondrial chromosome). The SNP with the highest minor allele frequency (MAF) was CNC10140681 (0.497), while the lowest was 8_129315758 (0.02), with an average MAF of 0.21. The SNP with the highest effect value was CNC10022746 (93.23), while the lowest was CNC10022920, with an average of 7.06. The SNP with the highest z-ratio was 8_129315758 (5.81), the lowest was 12_57934316 (−5.20), and the average was 2.61. *p*-value (*p*.value) ranged from a maximum of 1.96 × 10^−5^ to a minimum of 1.25 × 10^−8^, with an average of 3.3 × 10^−6^. Explained variance (expl.var) ranged from a maximum of 23.52 to a minimum of 4.38, with an average of 9.28 (Table 2).

### 3.3. Genome-Wide Variance Component Analysis

Genome-wide variance component analysis was conducted using ASReml 4.2 software to calculate model parameters including AIC, BIC, Aopt, logDopt, and heritability. Heritability encompassed both variance component-based heritability (vc-heritability) and PEV (percentage of explained variance)-based heritability (pev-heritability). The vc-heritability represents the generalized heritability at the genome-wide SNP level, defined as the proportion of genetic variance within the total phenotypic variance (Vp = Vg + Ve). It is calculated as h^2^ = Vg/(Vg + Ve). The pev-heritability represents the percentage of explained variance attributable to significant SNPs. This pev-heritability is estimated using the percentage of explained variance from principal component analysis (PCA) conducted during GWAS analysis.

An evaluation of heritability for 33 first-parity reproductive traits in Suzi sows revealed that effective vc-heritability was estimated for 17 traits, while effective pev-heritability was estimated for 21 traits. Among these, pev-heritability was also estimated for the 17 traits with vc-heritability estimates, showing a correlation coefficient of 0.29 between the two heritability estimates. Among these 17 traits, the mean pev-heritability (0.41) was higher than the mean vc-heritability (0.24). Specifically, vc-heritability for daily gain from birth to weaning (DGBW) was the highest, while pev-heritability for number of mummified piglets (NM) was the highest. Four traits (DGBW, NM, ACN, LCN) had vc heritabilities exceeding 0.5, classifying them as high-heritability traits. Seven traits had vc heritabilities between 0.1 and 0.3, six traits had vc heritabilities below 0.1, and twelve traits had vc heritabilities below 0.01 (Table 3).

There were ten traits significantly associated with SNPs in the GWAS. Their vc and pev heritabilities were as follows: AFS (<0.01, <0.01), AFF (<0.01, <0.01), NNB (0.21, 0.53), NH (<0.01, <0.01), NW (0.06, 0.48), NS (0.01, 0.45), NM (0.65, 0.7), ND (0.19, 0.53), PB (<0.01, <0.01), and CCN (<0.01, <0.01). The heritability of each trait, ranked from highest to lowest, was as follows: NM > NNB > ND > NW > NS (Table 3).

### 3.4. Gene Annotation

Significantly associated SNPs from the GWAS results for first-parity reproductive traits in Suzi sows, along with SNPs meeting different threshold values (5 × 10^−8^, 5 × 10^−6^, 5 × 10^−5^, 5 × 10^−4^, 5 × 10^−3^, 5 × 10^−2^), were annotated using the Ensembl database, MAGMA v1.10 software [17], Linux commands, R, and python programming. The gene annotation for each trait is summarized below: AFS (Figure 1) showed significant association with 10 SNPs, which were located on chromosomes 2, 3, 4, 8, 14, and 23, with chromosome 2 hosting the highest number of SNPs (4, accounting for 40%). Ranked by significance, the annotated genes for these SNPs were ADAMTS19, VIRMA, EPHA5, SERGEF, and PCDHAC2. AFF (Figure 2) was significantly associated with 9 SNPs, all of which were also significantly associated with AFS. Among these SNPs, 44.4% were located on chromosome 2. Ranked by significance, the annotated genes were ADAMTS19, EPHA5, PCDHAC2, SERGEF, and VIRMA.

NNB (Figure 3) showed significant association with 7 SNPs, 4 of which (57.14%) were located on chromosome 12, while others were on chromosomes 23 (X) and 24 (Y). None of these SNPs were annotated to any gene. NH (Figure 4) was significantly associated with only one SNP, CNC10131876, located on chromosome 13 and not annotated to any gene. NH (Figure 5) was significantly associated with six SNPs, five of which (83.33%) were located on chromosome 2, and one on chromosome 10. The annotated genes were ZNF354B, PROP1, CATSPERD, and HSD11B1L. NS (Figure 6) showed significant association with eight SNPs: four (50%) on chromosome 6, three (37.5%) on chromosome 4, and one on chromosome 15, annotated to genes DYNLRB2, HNF4G, and HSD17B2. NM (Figure 7) showed significant association with 17 SNPs, including 5 (29.41%) on chromosome 2, 3 (17.65%) on chromosomes 5 and 12. Ranked by significance, the annotated genes were as follows: LUZP2, AAAS, SLCO3A1-SV2B, KIF18A-BDNF, RARG, and KRT79. ND (Figure 8) showed significant association with 5 SNPs, including 2 (40%) on chromosome 7 and one each on chromosomes 3, 8, and 9. The annotated genes are PCARE and DICER1. PB (Figure 9) showed significant associations with 5 SNPs: 2 (40%) on chromosome 14, and one each on chromosomes 2, 13, and 16. No annotated genes were identified. CCN (Figure 10) showed association with only one SNP on chromosome 5, with no annotated gene identified.

In summary, these SNPs significantly associated with first-parity reproductive traits in Suzi sows annotated 20 candidate genes: ADAMTS19, VIRMA, EPHA5, SERGEF, PCDHAC2, EPHA5, ZNF354B, PROP1, CATSPERD, HSD11B1L, DYNLRB2, HNF4G, HSD17B2, LUZP2, AAAS, SLCO3A1-SV2B, KIF18A-BDNF, RARG, KRT79, PCARE, and DICER1. Some of these SNPs are located in gene regulatory regions, some in introns, and some in intergenic regions (Table 4).

### 3.5. Go Analysis of Annotated Genes

Annotated genes used for GO analysis include candidate genes and genes from GWAS results where *p* < 0.03 (lower *p*-values yielded no GO results). These genes were loaded into the R-studio platform. Then the following libraries were loaded onto the R-studio platform, including DOSE, enrichplot, ggplot2, org.Ss.eg.db, and topGO, etc. EnrichGO and related programs were executed to perform successive analyses of biological process (BP), molecular function (MF), and cellular component (CC). Results were visualized using four formats: dot plots, bar charts, network diagrams, and gene-concept networks. BP, MF, and CC analyses were displayed using dot plots, bar charts, gene-concept networks, and bar charts, respectively.

**Figure 11:**
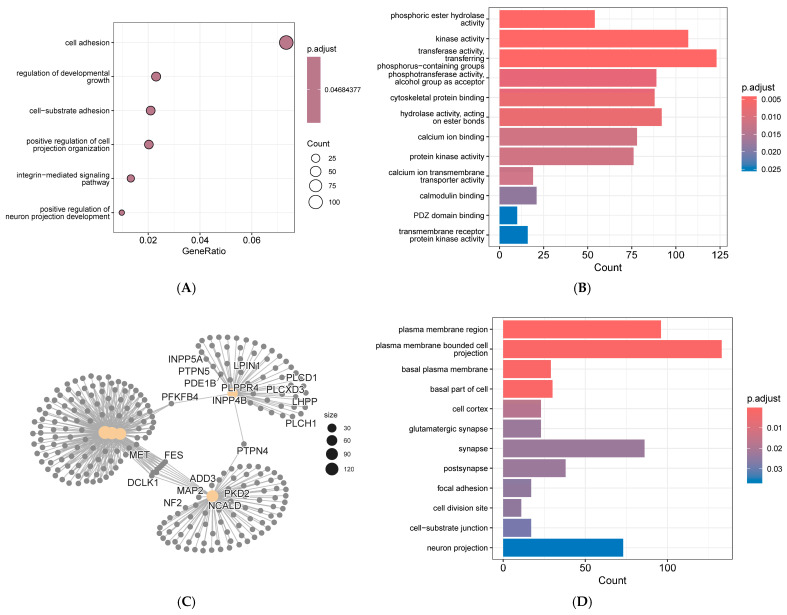
GO analysis results for annotated genes of first-parity reproductive traits in Suzi sows, (**A**) biological process dot plot, (**B**) molecular function bar chart, (**C**) molecular function gene-concept network diagram, (**D**) cellular component bar chart.

GO analysis revealed the top three enriched BPs were cell adhesion, positive regulation of cell projection organization, and positive regulation of neuron projection development. The top three enriched MFs were phosphoric ester hydrolase activity, kinase activity, and transferase activity, transferring phosphorus-containing groups. The top three enriched CC were plasma membrane region, plasma membrane-bounded cell projection, and basal plasma membrane (Figure 11).

### 3.6. Kegg Analysis of Annotated Genes

Annotated genes used for KEGG analysis include candidate genes and genes from GWAS results where *p* < 0.003 (lower *p*-values yielded no KEGG results). These genes were loaded into the R-studio platform. Then the following libraries were loaded onto the R-studio platform, including ClusterProfiler, DOSE, enrichplot, ggplot2, org.Ss.eg.db, Rgraphviz, and topGO. KEGG analysis was performed using the enrichKEGG function with pvalueCutoff = 0.05 and qvalueCutoff = 0.2. Results were visualized as dot plots, bar charts, and network diagrams.

**Figure 12:**
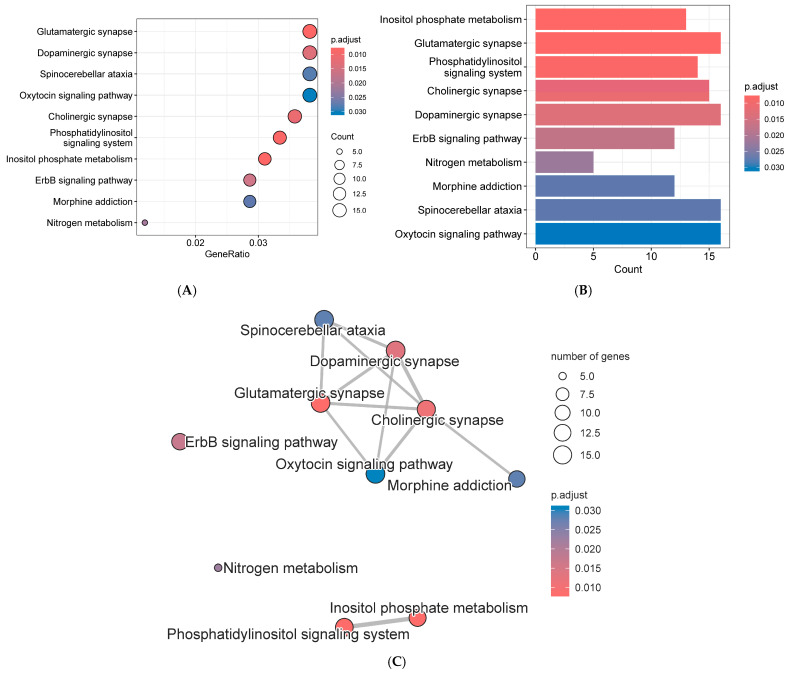
KEGG analysis results for annotated genes of first-parity reproductive traits in Suzi sows, (**A**) scatter plot, (**B**) bar chart, (**C**) network diagram.

KEGG analysis revealed that these genes collectively enriched 15 KEGG pathways. Sorted by *p*-value from lowest to highest, the categories were metabolism, organismal systems, environmental information processing, organismal systems, organismal systems, environmental information processing, metabolism, human diseases, human diseases, organismal systems, human diseases, environmental information processing, organismal systems, organismal systems, and organismal systems. The KEGG pathways were inositol phosphate metabolism, glutamatergic synapse, phosphatidylinositol signaling system, cholinergic synapse, dopaminergic synapse, erbb signaling pathway, nitrogen metabolism, morphine addiction, spinocerebellar ataxia, oxytocin signaling pathway, choline metabolism in cancer, hippo signaling pathway—multiple species, insulin secretion, parathyroid hormone synthesis, secretion and action, and gnrh secretion (Figure 12).

## 4. Discussion

### 4.1. Results of Reproductive Performance Assessment in First-Parity Sows

Results indicated that the average AFS was 345.36 d, and the average AFF was 459.17 d. This indicated that AFS and AFF for the experimental high-quality black sows were relatively high. However, the actual AFS could be significantly lower (as they reach sexual maturity early, typically around 200 d). This discrepancy may be attributed to the unique farrowing year, during which strict African Swine Fever (ASF) control measures were in place. Breeding was frequently suspended due to disease prevention requirements. Shiho Usui et al. (2015) [18] reported an initial breeding age of 280.5 d for purebred Berkshire sows and 240.7 d for Berkshire crossbred sows. SuHyup Lee et al. (2019) [19] studied 86 Yorkshire × Landrace crossbred sows and found no significant differences in litter size or weaned piglets across the first to fifth parities when initial breeding occurred at <220 d, 220–240 d, and >240 d. They proposed that AFS at 220 d and 140 kg body weight yields optimal production efficiency. In the study, the 2024 AFS is approaching this target.

The average TNB for first-parity sows was 9.79, with a maximum of 16. The average NBA was 8.9, the average number of non-viable piglets born (NNB) was 0.77, and the average number of stillborn piglets (NS) was 0.41. These results indicate that the TNB for first-parity Suzi sows was around 10, with 0.77 stillborn piglets, placing them at a disadvantage compared to commercial breeds. Ju et al. (2021) reported that first-parity sows exhibited TNB, NBA, and NS of 10.83, 10.59, and 0.16, respectively, in a population of 8420 healthy Landrace × Yorkshire hybrid sows with farrowing year 2017–2018 on a commercial pig farm in Central China [20].

The average weaning age (WA) for first-parity sows was 28.32 d, the average number of weaned piglets (NW) was 8.73, and the average individual weaning weight (IWW) was 6.55 kg. The average daily gain from birth to weaning (DGBW) was 207.19 g/d. This indicates a high survival rate of live piglets in the farrowing house (8.73/8.9 = 98.09%). Former study reported that using a total of 2184 pigs (DNA 600 × PIC L42), the average individual weaning weight (IWW) at 24.5 d was 6.18 kg. The average daily gain from birth to 42 d was 391 g/d [21]. This indicated that the IWW of Suzi sows in this study was slightly lower than those of crossbred sows (DNA 600 × PIC L42), and the DGBW in this study was slightly lower than those of crossbred sows.

Age at transfer to nursery (AN) was 34.86 d with an average weight of 8.61 kg, indicating accelerated growth (approximately 314.98 g/d) from post-weaning to nursery transfer (about 0–7 d after weaning). Archer et al. (2022) reported that an average daily gain (ADG) was 43 g/d during 0–7 d after weaning with IWW of 5.81 kg using 192 pigs [22]. This showed that the growth rate after weaning is significantly higher than before weaning in Suzi sows in this study.

### 4.2. Gwas Results for Reproductive Traits in First-Parity Sows

Among 33 first-parity farrowing traits of the Suzi pig, there were 10 traits significantly associated with genomic SNPs. These traits were as follows: age at first service (AFS), age at first farrowing (AFF), number of non-viable piglets born (NNB), number of healthy offspring (NH), number of weak offspring (NW), number of stillborn piglets (NS), number of mummified piglets (NM), number of deformed piglets (ND), percentage of pure black piglets (PB), and chest circumference at transfer to nursery (CCN). Among these traits, the SNPs significantly associated with AFS and AFF were largely consistent. Approximately 40% of these SNPs were located on chromosome 2, annotated to identical genes with different ranks by significance, and these genes were ADAMTS19, VIRMA, EPHA5, SERGEF, and PCDHAC2.

For trait NNB, 57.14% of SNPs were located on chromosome 12 with no annotated gene, while the sole SNP for trait NH was on chromosome 13 with no annotated gene. For trait NW, 83.33% of SNPs were on chromosome 2, annotated to the genes ZNF354B, PROP1, CATSPERD, and HSD11B1L. For trait NS, 50% of SNPs were located on chromosome 6, annotated to the genes DYNLRB2, HNF4G, and HSD17B2. For trait NM, 29.41% of SNPs were located on chromosome 2, annotated to the genes LUZP2, AAAS, SLCO3A1-SV2B, KIF18A-BDNF, RARG, and KRT79. In another study, a total of 816 litter records for trait NM were collected from 282 Landrace sows. GWAS results showed all of the detected SNPs were parity specific for NM in two breeds (Landrace and Large White). For trait NM, there were 7 SNPs on chromosome 2, and 4 SNPs on chromosome 12 in first-parity Landrace sows, and the candidate genes included OR2T6 and TAOK1 [23].

For trait ND, 40% of SNPs were located on chromosome 7, annotated to the genes PCARE and DICER1. For Trait PB, 40% of SNPs were located on chromosome 14 with no annotated gene, and for Trait CCN, the sole SNP was located on chromosome 5 with no annotated gene. Bhatia et al. (2013) analyzed six reproductive traits of TNB, NBA, NH, NW, NS, and NM from 516 Jinwu sows (crossbreeding of Jinhua and Duroc pigs). The results showed that a total of 771 genome-wide significant SNPs and 10 potential candidate genes associated with pig reproductive traits were identified: VOPP1, PGAM2, TNS3, LRFN5, ORC1, CC2D1B, ZFYYE9, TUT4, DCN, and FEZF1 [24]. In a total of 803 Duroc sows with 2807 farrowing records, reproductive traits of NBA, NM, and NS were associated with genomic SNPs, GWAS results showed that eight independent signals were ultimately identified, and there were seven promising candidate genes related to these traits, including ARID1A, RXRG, NFATC4, ABTB2, GRAMD1B, NDRG1, and APC [25]. The candidate genes associated with reproductive traits in this study are different from those in these published studies; the difference might be due to variations in pig breeds.

### 4.3. Genome-Wide Variance Component Analysis

Genome-wide variance component analysis was conducted for 33 first-parity reproductive traits in Suzi sows. Results showed that more traits had pev-heritability (21) than vc-heritability (17), and the correlation coefficient was 0.29 between pev-heritability and vc-heritability. Among these 17 traits, the mean pev-heritability (0.41) was higher than the mean vc-heritability (0.24). The vc-heritability for DGBW was the highest (0.7), while pev-heritability for NM was the highest (0.7).

In Suzi sows, the heritabilities (vc-heritability, pev-heritability) for commonly used pig reproductive traits were as follows: TNB (<0.01, <0.01), NBA (0.05, 0.48), AFS (<0.01, <0.01), NNB (0.21, 0.53), NS (0.01, 0.45), 0.65, 0.7, ND (0.19, 0.53), NH (<0.01, <0.01), NW (0.06, 0.48). Research indicated that in Large White pigs, the heritabilities (vc-heritability) for reproductive traits were as follows: TNB 0.02, NBA < 0.01, NNB 0.03, NS 0.07, NM < 0.01 [26]. Another research showed that using multi-breed data sets from Yorkshire, Landrace, and Duroc first-parity sows, the heritability of piglet mortality at birth was estimated to be 0.06 [27]. These findings illuminated that most genomic heritabilities of reproductive traits are relatively low, and most heritabilities are different between Suzi sows and commercial pigs.

### 4.4. Go and Kegg Analysis

For potential candidate genes of reproductive traits in first-parity Suzi sows, GO analysis results indicated that the top three enriched biological processes (BP) were cell adhesion, positive regulation of cell projection organization, and positive regulation of neuron projection development. The top three enriched molecular functions (MF) were phosphoric ester hydrolase activity, kinase activity, and transferase activity, transferring phosphorus-containing groups. The top three enriched cellular components (CC) were plasma membrane region, plasma membrane-bounded cell projection, and basal plasma membrane. For potential candidate genes of number of stillborn piglets (NS) in first-parity Duroc pigs, a total of 148 positional candidate genes were found and enriched in “GO: 0016485, protein processing”; “GO: 0006955, immune response”; “GO: 0007218, neuropeptide signaling pathway”; “GO: 0007155, cell adhesion”; and “GO: 0010950, positive regulation of endopeptidase activity” [25]. GO analysis of former research revealed the role of several biological processes and molecular functions like regulation of biological quality, growth, cell migration, steroid binding, etc., in reproductive traits of pigs when the embryo traverses the ampulla [28].

For potential candidate genes of reproductive traits in first-parity Suzi sows, KEGG analysis revealed that these genes were enriched in 15 KEGG pathways. Sorted by *p*-value from lowest to highest, the KEGG pathways were as follows: Inositol phosphate metabolism, glutamatergic synapse, phosphatidylinositol signaling system, cholinergic synapse, dopaminergic synapse, ErbB signaling pathway, Nitrogen metabolism, morphine addiction, spinocerebellar ataxia, oxytocin signaling pathway, choline metabolism in cancer, hippo signaling pathway—multiple species, insulin secretion, parathyroid hormone synthesis, secretion and action, and GnRH secretion. Research showed that using multi-breed data sets from Yorkshire, Landrace, and Duroc first-parity sows, there were six SNPs were observed in first parity, and the candidate genes found to associate with the reproductive system and embryonic development in the tissue expression database, which are reasonably related to piglet mortality [27].

## 5. Conclusions

In summary, this study conducted a GWAS analysis of first-parity reproductive traits in 33 Suzi pigs. Ultimately, 10 first-parity reproductive traits were significantly associated with 60 SNPs, further annotated to 20 reported candidate genes. These candidate genes showed GO enrichment related to cell adhesion, positive regulation of cell protrusion organization, and positive regulation of neuronal process development. KEGG enrichment was associated with inositol phosphate metabolism, glutamatergic synapse, ErbB signaling pathway, and the oxytocin signaling pathway.

## Figures and Tables

**Table 1:** Analysis results of 33 first-parity reproductive traits in Suzi sows used in the experiment.

Item	AFS	AFF	cEBV	GP	CNB	TNB
Average	345.36	459.17	100.43	113.8	9.34	9.79
Standard deviation	151.34	151.45	21.31	2.05	2.14	2.24
Item	NBA	NNB	NH	NW	NS	NM
Average	8.9	0.77	8.43	0.48	0.41	0.22
Standard deviation	2.26	1.19	2.08	0.93	0.85	0.62
Item	ND	mNBA	fNBA	LBW	IBW	NLT
Average	0.14	4.69	4.41	10.8	1.21	7.24
Standard deviation	0.53	1.87	1.55	3.03	0.26	0.44
Item	NRT	TNT	NP	PB	WA	NW
Average	7.26	14.51	3.92	76.24	28.32	8.73
Standard deviation	0.45	0.89	2.07	25.01	1.73	2.17
Item	IWW	LWW	DGBW	AN	BWN	BLN
Average	6.55	56.34	207.19	34.86	8.61	48.32
Standard deviation	1.21	17.39	37.68	12.97	6.05	4.84
Item	CCN	ACN	LCN			
Average	43.33	39.62	10.78			
Standard deviation	3.03	3.55	2.36			

Abbreviation meaning of each trait: age at first service (AFS), age at first farrowing (AFF), comprehensive estimated breeding value (cEBV), gestation period (GP), comprehensive number of piglets born (CNB), total number of piglets born (TNB), number of piglets born alive at birth (NBA), number of non-viable piglets born (NNB), number of healthy offspring (NH), number of weak offspring (NW), number of stillborn piglets (NS), number of mummified piglets (NM), number of deformed piglets (ND), number of male piglets born alive at birth (mNBA), number of female piglets born alive at birth (fNBA), litter birth weight (LBW), individual birth weight (IBW), number of left teats in piglets (NLT), number of right teats in piglets (NRT), total number of teats in piglets (TNT), number of spotted piglets (NP), percentage of pure black piglets (PB), weaning age (WA), number of weaned piglets (NW), individual weaning weight (IWW), litter weaning weight (LWW), daily gain from birth to weaning (DGBW), age at transfer to nursery (AN), body weight at transfer to nursery (BWN), body length at transfer to nursery (BLN), chest circumference at transfer to nursery (CCN), abdominal circumference at transfer to nursery (ACN), and leg circumference at transfer to nursery (LCN).

**Table 2:** GWAS parameters for SNPs significantly associated with first-parity reproductive traits in Suzi sows.

Trait	SNP	chr	pos	maf	Effect	*p*.Value	expl.var
age at first service (AFS)	CNC10022746	2	132,234,238	0.080607	92.95919	5.17 × 10^−8^	6.088653
	CNCB10003227	4	42,176,304	0.492991	64.73012	6.69 × 10^−7^	9.956982
	CNCB10001752	2	43,415,752	0.063084	91.56389	9.13 × 10^−7^	4.711177
	CNC10081297	8	64,117,040	0.133178	65.51755	1.07 × 10^−6^	4.711258
	CNC10230240	23	7304,150	0.432243	−62.55	1.14 × 10^−6^	9.128621
	CNCB10001743	2	41,306,798	0.364486	−63.3215	1.71 × 10^−6^	8.830169
	CNCB10002698	3	81,907,335	0.060748	90.11435	3.96 × 10^−6^	4.405144
	CNC10140681	14	34,388,947	0.496495	56.95693	4.18 × 10^−6^	7.710316
	CNC10022920	2	142,568,860	0.140187	−65.1442	4.80 × 10^−6^	4.863218
	CNC10032654	3	135,578,375	0.35514	−60.7004	7.48 × 10^−6^	8.022486
age at first farrowing (AFF)	CNC10022746	2	132,234,238	0.080607	93.22857	4.89 × 10^−8^	6.113294
	CNCB10003227	4	42,176,304	0.492991	64.87131	6.49 × 10^−7^	9.982999
	CNC10081297	8	64,117,040	0.133178	65.6649	1.03 × 10^−6^	4.724206
	CNCB10001752	2	43,415,752	0.063084	91.12987	1.05 × 10^−6^	4.658467
	CNC10230240	23	7,304,150	0.432243	−62.7986	1.06 × 10^−6^	9.185247
	CNCB10001743	2	41,306,798	0.364486	−63.0733	1.92 × 10^−6^	8.745775
	CNC10140681	14	34,388,947	0.496495	57.01878	4.17 × 10^−6^	7.713572
	CNCB10002698	3	81,907,335	0.060748	89.98522	4.18 × 10^−6^	4.384855
	CNC10022920	2	142,568,860	0.140187	−65.5027	4.36 × 10^−6^	4.908299
number of non-viable piglets born (NNB)	12_57934316	12	57,934,316	0.425754	−0.84146	3.17 × 10^−7^	23.52132
	X_3172285	23	3,172,285	0.441995	−0.83655	8.39 × 10^−7^	23.45181
	12_56124655	12	56,124,655	0.472158	0.748347	2.29 × 10^−6^	18.96438
	X_3176453	23	3,176,453	0.439675	−0.79842	2.41 × 10^−6^	21.33899
	12_56141636	12	56,141,636	0.470998	0.744105	3.16 × 10^−6^	18.74499
	Y_2815432	24	2,815,432	0.408353	−0.83261	3.64 × 10^−6^	22.75738
	12_56118044	12	56,118,044	0.472158	0.736089	4.69 × 10^−6^	18.34819
number of healthy offspring (NH)	CNC10131876	13	95,848,356	0.456977	1.018798	7.56 × 10^−6^	12.84277
number of weak offspring (NW)	CNC10021700	2	79,598,020	0.037123	0.843657	5.93 × 10^−7^	7.213283
	CNC10021702	2	79,632,416	0.037123	0.843657	5.93 × 10^−7^	7.213283
	CNC10021703	2	79,654,987	0.037123	0.843657	5.93 × 10^−7^	7.213283
	CNC10021544	2	73,221,843	0.035963	0.801703	2.75 × 10^−6^	6.317745
	CNC10021546	2	73,295,370	0.035963	0.801703	2.75 × 10^−6^	6.317745
	CNCB10007357	10	15,723,170	0.12181	0.434066	5.57 × 10^−6^	5.714374
number of stillborn piglets (NS)	CNC10060156	6	7,647,082	0.091647	0.534188	1.93 × 10^−6^	5.945918
	4_60598574	4	60,598,574	0.091647	0.543139	2.68 × 10^−6^	6.146847
	4_60633363	4	60,633,363	0.091647	0.543139	2.68 × 10^−6^	6.146847
	4_60143356	4	60,143,356	0.054524	0.636295	2.83 × 10^−6^	5.224121
	CNC10060142	6	7,132,347	0.096288	0.516031	3.71 × 10^−6^	5.79974
	CNC10152171	15	109,828,092	0.063805	0.628791	7.54 × 10^−6^	5.911394
	CNCB10004308	6	6,241,186	0.430394	0.401679	7.76 × 10^−6^	9.900437
	CNCB10004309	6	6,317,896	0.430394	0.401679	7.76 × 10^−6^	9.900437
number of mummified piglets (NM)	2_35090057	2	35,090,057	0.022042	0.856174	6.46 × 10^−7^	7.81605
	2_35184348	2	35,184,348	0.022042	0.856174	6.46 × 10^−7^	7.81605
	2_35304516	2	35,304,516	0.022042	0.856174	6.46 × 10^−7^	7.81605
	10_1094605	10	1,094,605	0.082367	0.493245	7.79 × 10^−7^	9.095816
	5_18526734	5	18,526,734	0.026682	0.836982	1.23 × 10^−6^	8.999204
	7_87309677	7	87,309,677	0.075406	0.508977	1.41 × 10^−6^	8.934071
	2_32394198	2	32,394,198	0.465197	0.402239	1.63 × 10^−6^	19.91114
	11_55264554	11	55,264,554	0.058005	0.576168	1.73 × 10^−6^	8.972348
	1_608863	26	608,863	0.056845	0.555878	2.17 × 10^−6^	8.194593
	12_55351712	12	55,351,712	0.023202	0.80172	3.02 × 10^−6^	7.205587
	12_55045321	12	55,045,321	0.023202	0.775936	3.86 × 10^−6^	6.749571
	12_55094012	12	55,094,012	0.023202	0.775936	3.86 × 10^−6^	6.749571
	5_18448981	5	18,448,981	0.054524	0.544356	3.91 × 10^−6^	7.556202
	7_88172698	7	88,172,698	0.053364	0.561383	4.93 × 10^−6^	7.874957
	11_56289609	11	56,289,609	0.038283	0.609406	5.37 × 10^−6^	6.763383
	2_34903944	2	34,903,944	0.423434	−0.44095	6.96 × 10^−6^	23.48065
	5_18115698	5	18,115,698	0.037123	0.582897	7.68 × 10^−6^	6.007498
number of deformed piglets (ND)	8_129315758	8	129,315,758	0.020882	0.666854	1.25 × 10^−8^	8.789016
	3_110221403	3	110,221,403	0.051044	0.394235	6.00 × 10^−7^	7.277446
	CNC10072333	7	116,430,733	0.093968	0.279291	3.65 × 10^−6^	6.419684
	CNC10070593	7	27,389,510	0.055684	0.358172	4.58 × 10^−6^	6.520967
	9_5732524	9	5,732,524	0.047564	0.370071	4.92 × 10^−6^	5.997376
percentage of pure black piglets (PB)	CNC10020510	2	23,172,010	0.487952	−11.0069	1.5 × 10^−6^	10.35707
	CNCB10010204	14	131,325,841	0.46988	−10.773	5.25 × 10^−6^	9.891355
	CNC10130116	13	6,000,732	0.457831	9.161748	6.9 × 10^−6^	7.128846
	CNCB10010264	14	140,199,680	0.445783	10.00824	7.02 × 10^−6^	8.467228
	CNCB10011174	16	233,939	0.451807	−8.96875	7.05 × 10^−6^	6.816688
chest circumference at transfer to nursery (CCN)	CNC10051999	5	105,361,021	0.091667	−3.20897	1.96 × 10^−5^	18.98266

Note: maf (minor allele frequency) means the frequency of the minor allele, effect means the degree to which a specific SNP influences a particular trait; z.ratio measures the strength of association between a specific SNP and a particular phenotype; expl.var means the degree to which a SNP accounts for phenotypic variation.

**Table 3:** Genome-wide variance component of heritability for first-parity reproductive trait in Suzi sows.

Number	Trait	vc-Heritability	pev-Heritability
1	age at first service (AFS)	<0.01	<0.01
2	age at first farrowing (AFF)	<0.01	<0.01
3	comprehensive estimated breeding value (cEBV)	0.21 ± 0.07	0.53
4	gestation period (GP)	0.1 ± 0.07	0.49
5	comprehensive number of piglets born (CNB)	<0.01	<0.01
6	total number of piglets born (TNB)	<0.01	<0.01
7	number of piglets born alive at birth (NBA)	0.05 ± 0.06	0.48
8	number of non-viable piglets born (NNB)	0.21 ± 0.07	0.53
9	number of healthy offspring (NH)	<0.01	<0.01
10	number of weak offspring (NW)	0.06 ± 0.06	0.48
11	number of stillborn piglets (NS)	0.01 ± 0.07	0.45
12	number of mummified piglets (NM)	0.65 ± 0.1	0.7
13	number of deformed piglets (ND)	0.19 ± 0.07	0.53
14	number of male piglets born alive at birth (mNBA)	<0.01	<0.01
15	number of female piglets born alive at birth (fNBA)	0.03 ± 0.06	0.05
16	litter birth weight (LBW)	0.01 ± 0.09	0.4
17	individual birth weight (IBW)	0.01 ± 0.08	0.41
18	number of left teats in piglets (NLT)	<0.01	0.47
19	number of right teats in piglets (NRT)	<0.01	0.47
20	total number of teats in piglets (TNT)	<0.01	<0.01
21	number of spotted piglets (NP)	0.33 ± 0.12	0.27
22	percentage of pure black piglets (PB)	<0.01	<0.01
23	weaning age (WA)	<0.01	<0.01
24	number of weaned piglets (NW)	<0.01	<0.01
25	individual weaning weight (IWW)	<0.01	<0.01
26	litter weaning weight (LWW)	<0.01	0.03
27	daily gain from birth to weaning (DGBW)	0.7 ± 0.34	0.45
28	age at transfer to nursery (AN)	0.12 ± 0.12	0.12
29	body weight at transfer to nursery (BWN)	<0.01	0.03
30	body length at transfer to nursery (BLN)	0.3 ± 0.14	0.24
31	chest circumference at transfer to nursery (CCN)	<0.01	<0.01
32	abdominal circumference at transfer to nursery (ACN)	0.59 ± 0.13	0.43
33	leg circumference at transfer to nursery (LCN)	0.59 ± 0.13	0.43

**Table 4:** Gene annotations for SNPs significantly associated with first-parity reproductive traits in GWAS analysis.

Trait	SNP	GenBank	SNP_Ensembl
age at first service (AFS)	CNC10022746	ADAMTS19	intron_variant
	CNCB10003227	VIRMA	intron_variant
	CNC10081297	EPHA5	intergenic_region
	CNCB10001743	SERGEF	intron_variant
	CNC10022920	PCDHAC2	intron_variant
age at first farrowing (AFF)	CNC10022746	ADAMTS19	intron_variant
	CNCB10003227	VIRMA	intron_variant
	CNC10081297	EPHA5	intergenic_region
	CNCB10001743	SERGEF	intron_variant
	CNC10022920	PCDHAC2	intron_variant
number of weak offspring (NW)	CNC10021700	ZNF354B	intron_variant
	CNC10021702	PROP1	upstream_gene_variant
	CNC10021544	CATSPERD	intron_variant
	CNC10021546	HSD11B1L	upstream_gene_variant
number of stillborn piglets (NS)	CNC10060156	DYNLRB2	downstream_gene_variant
	4_60598574	HNF4G	intron_variant
	4_60633363	HNF4G	intron_variant
	CNCB10004309	HSD17B2	intron_variant
number of mummified piglets (NM)	2_35090057	LUZP2	intron_variant
	2_35184348	LUZP2	intron_variant
	2_35304516	LUZP2	intron_variant
	5_18526734	AAAS	synonymous_variant
	7_87309677	SLCO3A1-SV2B	intergenic_region
	2_32394198	KIF18A-BDNF	intergenic_region
	5_18448981	RARG	upstream_gene_variant
	5_18115698	KRT79	intron_variant
number of deformed piglets (ND)	3_110221403	PCARE	downstream_gene_variant
	CNC10072333	DICER1	intron_variant

## Data Availability

The original contributions presented in this study are included in the article. Further inquiries can be directed to the corresponding author.

## Abbreviations

The following abbreviations are used in this manuscript:

GWAS	Genome-wide association study
SNP	Single-nucleotide polymorphism
QTL	Quantitative trait loci
AFS	Age at first service
AFF	Age at first farrowing
cEBV	Comprehensive estimated breeding value
GP	Gestation period
CNB	Comprehensive number of piglets born
TNB	Total number of piglets at birth
NBA	Number of piglets born alive at birth
NNB	Number of non-viable piglets born
NH	Number of healthy offspring
NW	Number of weak offspring
NS	Number of stillborn piglets
NM	Number of mummified piglets
ND	Number of deformed piglets
mNBA	Number of male piglets born alive at birth
fNBA	Number of female piglets born alive at birth
LBW	Litter birth weight
IBW	Individual birth weight
NLT	Number of left teats in piglets
NRT	Number of right teats in piglets
TNT	Total number of teats in piglets
NP	Number of spotted piglets
PB	Percentage of pure black piglets
WA	Weaning age
NW	Number of weaned piglets
IWW	Individual weaning weight
LWW	Litter weaning weight
DGBW	Daily gain from birth to weaning
AN	Age at transfer to nursery
BWN	Body weight at transfer to nursery
BLN	Body length at transfer to nursery
CCN	Chest circumference at transfer to nursery
ACN	Abdominal circumference at transfer to nursery
LCN	Leg circumference at transfer to nursery
TNB	Total number of piglets born
NBA	Number of born piglets alive
BP	Biological process
MF	Molecular function
CC	Cellular component
